# Automated myocardium segmentation in late gadolinium enhanced MR images

**DOI:** 10.1186/1532-429X-16-S1-P346

**Published:** 2014-01-16

**Authors:** Qian Tao, Rob J van der Geest

**Affiliations:** 1 Leiden University Medical Center, Leiden, Netherlands

## Background

Late Gadolinium Enhanced (LGE) MRI has proven clinical value for diagnosis and prognosis of post-infarct patients. A prerequisite for accurate myocardial scar assessment is the reliable segmentation of the myocardium. However, in post-infarct patients, the myocardial scar is often connected to the blood pool, while the contrast between them is typically poor. The ambiguous border between myocardial scar and blood pool substantially complicates manual contouring, and potentially gives rise to over- or under-estimation of the scar.

## Methods

We propose an automated LGE segmentation algorithm, which utilizes the myocardium morphology information from the cine sequence in the same study. In practice, the cine contours of the end-diastolic and end-systolic phases are routinely annotated for functional analysis. From the available cine contours, the potential endo- and epicardial contour sets for each LGE slice were estimated by cine-LGE registration and linear interpolation. Secondly, the match between the dual contour sets and each LGE slice was evaluated by correlating the LGE slice with the contour image constituted of edge filters adaptive to the edge direction. Finally, the global optimal contour was established by dynamic programming through the entire LGE stack. The method was validated on the LGE MR of 30 post-infarct patients with an in-plane resolution of 1.56 mm, and compared to the manual contouring results.

## Results

**Figure 1:**
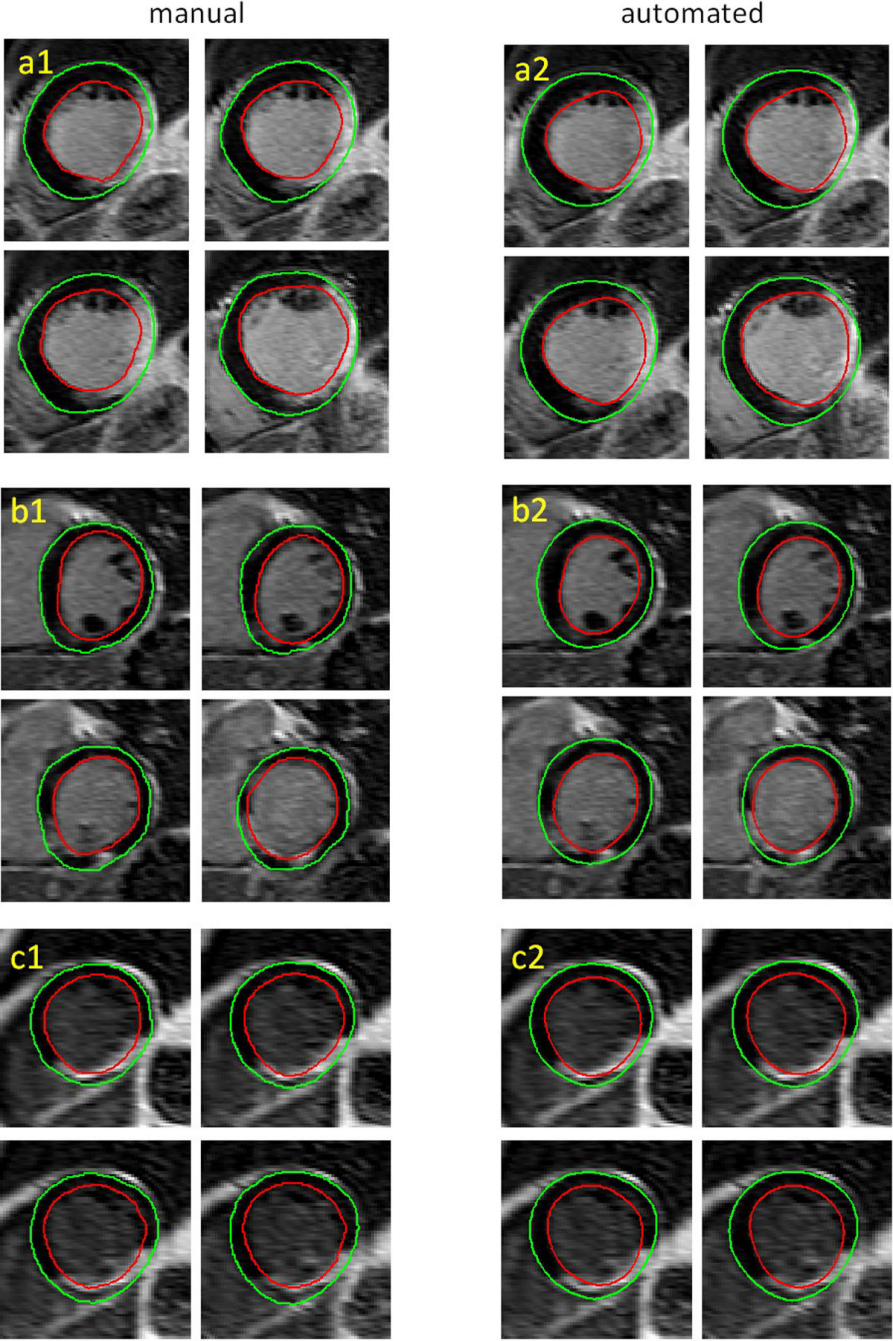
**Comparison between the manual and automated LGE segmentation in 3 examples with different scar distribution and blood-scar contrast**. Left column (a1,b1,c1): manual LGE segmentation. Right column (a2,b2,c2): automated LGE segmentation.

The proposed method achieved sub-pixel accuracy in comparison to manual segmentation from experienced observers (Figure 1). The distances between the automated contours and manual contours were 1.0 ± 0.8 pixels (median 0.8) for the endocardial contour, and 0.8 ± 0.7 pixels (median 0.6) for the epicardial contour. The Dice overlap index between the segmented myocardium regions was 0.83 ± 0.03. The Full-Width-Half-Maxima method was applied to identify the myocardial scar from the segmented myocardium region. Myocardial scar size derived from the manual contours was comparable to that from the automated contours: 23.8 ± 16.8 ml and 22.7 ± 15.4 ml (p = NS), with Pearson's correlation 0.87 (p < 0.0001).

## Conclusions

We proposed an automated method to segment the myocardium in LGE for myocardial scar assessment. The method resulted in comparable performance with manual scar segmentation by experienced observers, in terms of contouring accuracy and myocardial scar size. The method does not require extra cine contour analysis than routinely performed, and reduces the observer-dependency by avoiding visual interpretation of LGE.

## Funding

The work was financially supported by the EU MEDIATE project (ITEA2-09039).

